# Blockade of growth hormone secretagogue receptor 1A signaling by JMV 2959 attenuates the NMDAR antagonist, phencyclidine-induced impairments in prepulse inhibition

**DOI:** 10.1007/s00213-015-4054-3

**Published:** 2015-08-29

**Authors:** Jörgen A. Engel, Elisabet Jerlhag, Lennart Svensson, Roy G. Smith, Emil Egecioglu

**Affiliations:** Department of Pharmacology, Institute of Neuroscience and Physiology, The Sahlgrenska Academy at the University of Gothenburg, Medicinaregatan 13A, SE-405 30 Gothenburg, Sweden; Department of Metabolism and Aging, Scripps Research Institute-Florida, Jupiter, FL USA; Institute of Experimental Medical Sciences, Lund University, Lund, Sweden

**Keywords:** Cognition, Dopamine, Glutamate, NMDA

## Abstract

**Rationale:**

Schizophrenic-spectrum patients commonly display deficits in preattentive information processing as evidenced, for example, by disrupted prepulse inhibition (PPI), a measure of sensorimotor gating. Similar disruptions in PPI can be induced in rodents and primates by the psychotomimetic drug phencyclidine (PCP), a noncompetitive inhibitor of the NMDA receptor. Mounting evidence suggests that the hunger hormone ghrelin and its constitutively active receptor influences neuronal circuits involved in the regulation of mood and cognition.

**Objectives:**

In the present series of experiments, we investigated the effects of ghrelin and the growth hormone secretagogue receptor (GHS-R1A) neutral antagonist, JMV 2959, on acoustic startle responses (ASR), PPI, and PCP-induced alterations in PPI.

**Results:**

Intraperitoneal (i.p.) administration of ghrelin (0.033, 0.1, and 0.33 mg/kg) did not alter the ASR or PPI in rats. Conversely, i.p. injection of JMV 2959 (1, 3, and 6 mg/kg), dose dependently decreased the ASR and increased PPI. Pretreatment with JMV 2959 at a dose with no effect on ASR or PPI per se, completely blocked PCP-induced (2 mg/kg) deficits in PPI while pretreatment with the highest dose of ghrelin did not potentiate or alter PPI responses of a sub-threshold dose of PCP (0.75 mg/kg).

**Conclusion:**

These findings indicate that the GHS-R1A is involved in specific behavioral effects of PCP and may have relevance for patients with schizophrenia.

## Introduction

The growth hormone secretagogue receptor (GHS-R1A), initially an orphan receptor activated by growth hormone-releasing peptides and nonpeptidyl ligands such as GHRP-6 and MK-0677, is expressed in discrete areas throughout the central nervous system (Howard et al. 1996; Guan et al. 1997). The receptor, which mediates several biological activities, including secretion of GH and stimulation of appetite and serves to maintain energy homeostasis, is constitutively active when expressed in cell lines and is activated by its endogenous gastric-derived ligand ghrelin (Howard et al. 1996; Kojima et al. 1999; Holst et al. 2003). In recent years, there has been an increasing interest in the modulatory effect of ghrelin and the GHS-R1A on central dopamine and glutamate signaling (Abizaid et al. 2006; Jerlhag et al. 2006, 2011a, b; Jiang et al. 2006; Kern et al. 2012; Goshadrou et al. 2013; Ghersi et al. 2014). Ghrelin and GHSR-1A ligands, thus, have been shown to regulate feeding behavior, memory function, and cognition via dopamine and/or glutamate signaling (Egecioglu et al. 2010; Jacoby and Currie 2011; Jerlhag et al. 2011a; Goshadrou et al. 2013; Ghersi et al. 2014). Furthermore, ghrelin augments while GHSR-R1A antagonist attenuate cocaine- and amphetamine-induced locomotor stimulation and accumbal dopamine release (Wellman et al. 2008; Jerlhag et al. 2010) as well as the rewarding properties of alcohol (Jerlhag et al. 2009) consistent with effects on dopamine signaling.

Patients with psychiatric disease, in particular schizophrenia-spectrum patients, are commonly unable to filter incoming sensory stimuli, which led to the hypothesis that these patients are afflicted by impairments in information processing (Braff et al. 1978; Freedman et al. 1987; Braff 1993). Deficits in preattentive information processing/gating mechanisms, as measured for example by prepulse inhibition (PPI) of the acoustic startle reflex, are found in patients with psychiatric disease (Braff et al. 1978). Alterations in PPI responses has also been demonstrated following the administration of various psychotomimetic drugs affecting central dopaminergic and glutamatergic signaling, such as amphetamine in humans and rodents (Mansbach et al. 1988; Hutchison and Swift 1999) or the *N*-methyl-d-aspartate (NMDA)-receptor antagonists phencyclidine (PCP) in monkeys and rodents (Bakshi et al. 1994; Javitt and Lindsley 2001). In contrast to the effects of PCP on PPI responses in rodents and monkeys, other NMDA receptor antagonists, such as ketamine and memantine, increase PPI responses when tested in humans (Duncan et al. 2001; Swerdlow et al. 2009). In humans, PCP mimics the symptomology of schizophrenia in the sense that it encompasses both negative and positive symptoms as well as cognitive dysfunctions (Allen and Young 1978). Phencyclidine also causes behavioral abnormalities in experimental animals that are similar to those observed in patients with schizophrenia (Moghaddam and Adams 1998). PCP-induced deficit in sensorimotor gating has been shown to be antagonized by both atypical antipsychotics such as clozapine (Bakshi et al. 1994) and recently also by the new dopamine stabilizer aripirazole (Fejgin et al. 2007), underlining the interaction between dopaminergic and glutamatergic signaling in schizophrenia.

Given the dopamine modulatory effects of ghrelin/GHSR-1A signaling combined with the neuroanatomical overlap found between the central expression of the GHSR-1A and areas recognized to be involved in sensorimotor gating (Guan et al. 1997; Swerdlow et al. 2001), prompted us to investigate the involvement of GHS-R1A and ghrelin signaling on NMDA receptor-mediated deficits in prepulse inhibition, a model of schizophrenia, in rodents.

## Materials and methods

### Animals

Two-hundred-gram male Sprague–Dawley rats (B & K Universal AB, Sollentuna, Sweden) were used in the study. Upon arrival, the animals were housed in groups of four and allowed to acclimatize for 1 week before the start of the experiment. They were maintained under a 12/12-h light/dark cycle (lights on at 0600 hours), constant humidity (50 %), and temperature (20 ± 1 °C) and had free access to standard food pellet (Lactamin, Vadstena, Sweden) and tap water. The study was approved by the local Ethics Committee at the University of Gothenburg, Sweden.

### Drugs, doses, and administration

All drugs used were dissolved in a physiological saline solution (0.9 % NaCl) in the morning on the day of the experiment and administered in a volume of 2 ml/kg via intra peritoneal (i.p.) injections. Acyl ghrelin (Tocris, Bristol, UK) was given in a dose range (0.033, 0.1, and 0.33 mg/kg) that previously has been shown to affect feeding responses, central c-Fos expression, and behavior (Hewson and Dickson 2000; Wren et al. 2000; Davis et al. 2007). The doses of the selective GHSR-1A neutral antagonist, JMV 2959 (a gift from AeternaZentaris GmBH, Frankfurt, Germany), used for the JMV 2959 dose response were 1, 3, and 6 mg/kg. The 3 and 6 mg/kg doses have previously been shown to inhibit ghrelin and fasting-induced feeding and affect various behavioral responses (Salome et al. 2009). Phencyclidine hydrochloride (PCP, Sigma, St. Louis, MO, USA) was given at a dose of 2 mg/kg, which is known to produce robust disruptions of PPI (Geyer et al. 2001). The sub-threshold dose of PCP used was 0.75 mg/kg which has no or very weak effects on PPI (Geyer et al. 2001). For the interaction studies between PCP and JMV 2959 or ghrelin, a dose of 2 mg/kg of JMV 2959 and 0.33 mg/kg of ghrelin was used.

### Prepulse inhibition apparatus

Acoustic startle was recorded using a MOPS 3 startle response recording system (Metod och Product Svenska AB, Sweden). The animals were placed in small Plexiglas® cages (10 × 5.5 × 6 cm) that were suspended at the top in a piston. The movements of the animal in the cage were registered by a piezo-electric accelerometer connected to the piston, and the signal generated was digitized by a microcomputer that also controlled the delivery of acoustic stimuli. Startle amplitude was defined as the maximum signal amplitude occurring 8–30 ms after the startle-eliciting stimulus, hence taking response latency into account. Four cages were used simultaneously and each cage was housed in a dimly lit and sound-attenuated cabinet (52 × 42 × 38 cm). The cages were calibrated for equal sensitivity prior to testing and each animal was always tested in the same cage at subsequent tests in order to minimize intertrial variation. The acoustic stimuli consisted of white noise, which was delivered by two high-frequency loudspeakers built into the ceiling of the cabinet.

### Prepulse inhibition paradigm

Each test session was initiated with an 8-min adaptation period containing only white background noise at 62 dB followed by series of five startle pulse-alone trials and five prepulse-alone trials. These initial pulse-alone trials served only to accommodate the animals to the sudden change in stimulus conditions and were omitted from the data analysis and the prepulse-alone trials were analyzed to ensure that they did not evoke any startle responses on their own. The animals were then subjected to a pseudo-randomized combination of three prepulse-alone trials for each prepulse intensity, 45 pulse-alone trials and 15 prepulse-pulse trials for each of the three prepulse intensities. Trials were separated by 5- to 15-s intervals and the test sessions lasted approximately 24 min including the adaptation period. The startle pulse was set to 105 dB and prepulse intensities to 9, 12, and 15 dB above background. Duration of acoustic stimuli was set to 20 ms for both prepulses and startle pulses and the interstimulus interval was set to 40 ms.

### Experimental design

All animals used in the experiments were initially subjected to a pretest in the startle apparatus without drug treatment to ensure that they expressed basal startle activity and PPI. Animals with deviant acoustic startle response (ASR) or PPI in the pretest were excluded from the experiments. Prior to all sessions, the animals were put in the test room in the morning at least 1 h prior to the test in order to habituate them to the test environment.

### Experiment 1: JMV 2959 dose response

The animals (*n* = 15) were randomly assigned to an initial treatment dose or vehicle and subsequently received all the different doses tested in a counter balanced design. Each test was separated by a 3- to 4-day-long washout period. The rats were given the injection of JMV 2959 (or vehicle) 17 min prior to being placed in the startle cages (i.e., 25 min prior to the first pulse).

### Experiment 2: JMV 2959 in combination with PCP

In order to examine the putative interaction between JMV 2959 and PCP, rats (*n* = 23) were pretreated with JMV 2959 (2 mg/kg) or vehicle 10 min prior to the injection of PCP (2 mg/kg) or vehicle. Seven minutes following the last injection, the animals were placed in the startle cages for the adaptation period and subsequent PPI testing. Each animal received all of the four treatment combinations (sal/sal, JMV2959/sal, sal/PCP, and JMV2959/PCP) in a counter-balanced design. Each test was separated by a 3- to 4-day-long washout period.

### Experiment 3: ghrelin dose response

The ghrelin dose response test was performed in the same way as experiment 1 except the animals (*n* = 24) received ghrelin injections.

### Experiment 4: ghrelin in combination with low-dose PCP

Animals (*n* = 12 in each group) were assigned to one of the following four treatment combinations: sal/sal, ghrelin/sal, sal/PCP, and ghrelin/PCP. The animals were first pretreated with ghrelin (0.33 mg/kg) or vehicle (25 min prior to first pulse) and 10 min later injected with either PCP (0.75 mg/kg) or vehicle. Seven minutes following the last injection, the animals were placed in the startle cages for adaption and subsequent PPI testing.

### Data and statistical analysis

The mean response amplitude for pulse-alone trials (P) was calculated for each test. This measure was used in the statistical analysis to assess drug-induced changes in acoustic startle response (ASR). The mean response amplitude for prepulse-pulse trials (PP) was also calculated and used to express the prepulse inhibition (PPI) according to the following formula:

$$ \mathrm{P}\mathrm{P}\mathrm{I}\left(\%\right)=100-\left[\left(\mathrm{P}\mathrm{P}/\mathrm{P}\right)\ast 100\right] $$

Experiments 1 and 2 were analyzed by a two-way repeated measures ANOVA with treatment dose and prepulse intensity as within-subject factors. A three-way mixed model ANOVA with pretreatment and treatment as between-subject factors and prepulse intensity as within-subject factor was applied when analyzing data from experiment 3 while experiment 4 was analyzed using a three-way repeated measures ANOVA with pretreatment, treatment, and prepulse intensity as within-subject factors. There was a significant main effect of prepulse intensity in each experiment (data not shown); however, as no prepulse intensity × pretreatment × treatment interaction was obtained (i.e., the effect of prepulse intensity did not vary significantly between testing conditions), PPI data collapsed across prepulse intensities and presented as an average %PPI throughout. The acoustic startle response and intertrial activity (ITA) were analyzed using a two-way repeated measures or mixed model ANOVA with pretreatment and treatment as within- or between-subject factors depending on the experiment type. A Bonferroni post hoc analysis was done to compare individual treatment combinations or doses.

## Results

Using prepulse noise level (9, 12, or 15 dB above background level) and the different treatment combinations or doses as within-subject factors revealed no statistically significant interaction between treatments and noise level for the dose response studies and the JMV 2959/PCP interaction study (ghrelin dose response; *F*(6,138) = 1.05, ns; JMV 2959 dose response: *F*(6,84) = 2.37, ns; JMV 2959/PCP: *F*(2,44) = 1.40, ns)). Furthermore, the interaction study investigating the possible influence of ghrelin treatment on sub-threshold PCP revealed no statistically significant interactions between noise level and treatment (ghrelin/PCP; *F*(2,76) = 2.9, ns). Consequently, changes in prepulse level were considered not to significantly alter the effect of treatment on PPI and hence noise levels were collapsed across intensities and the resultant variable was used in the statistical analysis.

**Fig. 1:**
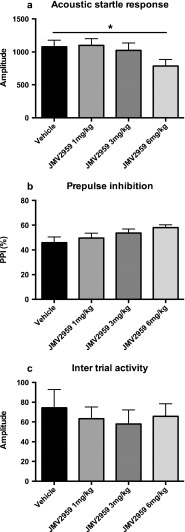
Effects of increasing doses of the ghrelin antagonist JMV 2959 (1–6 mg/kg, i.p.) on acoustic startle (**a**), prepulse inhibition of acoustic startle (**b**), and intertrial activity (**c**). JMV 2959 was injected 25 min before the first pulse. The data are presented as mean values ± SEM. *p < 0.05 compared to saline treatment (statistically significant ANOVA followed by Bonferroni post hoc test)

Treatment with the ghrelin antagonist, JMV 2959 dose dependently decreased the startle response (*F*(3.42) = 4.4, *p* < 0.01) and increased %PPI (*F*(3.42) = 3.9, *p* < 0.05) in the prepulse inhibition paradigm. The alteration in the startle response was mainly due to a 27 % decrease in the startle seen in the highest dose of JMV 2959 (6 mg/kg) compared to vehicle (*p* < 0.05, Bonferroni post hoc test) (Fig. 1a). Even though the ANOVA revealed a dose-dependent increase in the %PPI response, no differences between individual JMV 2959 treatment doses or vehicle could be found (Fig. 1b). No overall difference in intertrial activity was found in the ANOVA (*F* (3,42) = 0.44, ns) (Fig. 1c).

**Fig. 2:**
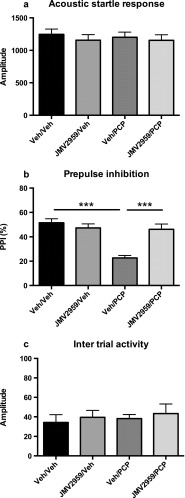
Interaction between JMV 2959 (2 mg/kg, i.p.) and PCP (2 mg/kg, i.p.) on acoustic startle (**a**), prepulse inhibition of acoustic startle (**b**), and intertrial activity (**c**). JMV 2959 was injected 25 min and PCP injected 15 min before the first pulse. The rats were tested every 3–4 days in a randomized order until they had received all treatments. The data are presented as mean values ± SEM. ***p < 0.001 (statistically significant ANOVA followed by Bonferroni post hoc test)

In order to investigate the possible interaction between GHSR-1A signaling and PCP-induced disruptions in PPI, animals were pretreated with a dose of JMV 2959 (2 mg/kg) in the dose range with no effect per se on startle response or PPI, prior to PCP treatment. A significant interaction between JMV 2959 and PCP treatment was found in the ANOVA (*F*(1,22) = 26.3, *p* < 0.001). JMV2959/sal treatment had no effect on %PPI (*p* > 0.05) while sal/PCP treatment induced a 57 % decrease in %PPI (*p* < 0.001, Bonferroni post hoc test, Fig. 2b). Pretreatment with JMV 2959 completely reversed the effect of PCP (Fig. 2b). The ANOVA revealed no effects on startle response (*F*(1,22) = 1.3, ns; Fig. 2a) or intertrial activity (*F*(1,22) = 1.5, ns; Fig. 2c) by PCP treatment or pretreatment with JMV.

**Fig 3:**
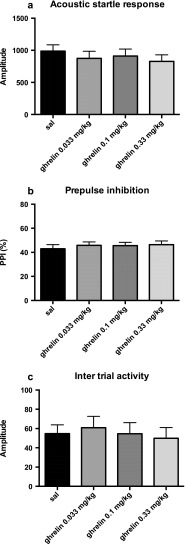
Effects of increasing doses of the ghrelin (0.033, 0.1, and 0.33 mg/kg, i.p.) on acoustic startle (**a**), prepulse inhibition of acoustic startle (**b**), and intertrial activity (**c**). Ghrelin was injected 25 min before the first pulse. The data are presented as mean values ± SEM

An ANOVA analysis of the ghrelin dose response did not reveal any overall alterations in startle (*F*(3,69) = 1.74, ns), PPI response (*F*(3,69) = 0.46, ns), or intertrial activity (*F*(3,69) = 0.78, ns. Fig. 3a–c). Furthermore, the highest dose of ghrelin used (0.33 mg/kg) did not potentiate or alter the effect of a sub-threshold dose of PCP on any of the measured outcomes (startle (*F*(1,38) = 0.73, ns), PPI% (*F*(1,38) = 0.32, ns), intertrial activity (*F*(1,38) = 1.66, ns) (data not shown).

## Discussion

Herein, we show that modulation of the GHS-R1A alter acoustic startle responses (ASR) as well as prepulse inhibition (PPI) of the ASR. Specifically, JMV2959, a highly selective GHSR-1A antagonist, dose dependently decreased ASR and increased %PPI. In addition, JMV 2959 completely blocked the effects of PCP-induced deficits in PPI at a dose that by itself did not significantly affect either ASR or PPI. On the contrary, peripheral treatment with ghrelin did not have any effect on ASR or PPI and did not potentiate PCP-induced effects on PPI.

Recent findings has shown that modulation of the GHS-R1A signaling alters dopamine release and dopamine turnover in both subcortical and prefrontal areas of the brain and that antagonism at the GHS-R1A can block dopamine release in response to drugs of abuse. Our finding that JMV 2959 dose dependently increase %PPI and decrease ASR in animals could possibly be explained by the modulatory effects of ghrelin and GHS-R1A signaling on dopamine transduction. Interestingly, heterodimerization of GHS-R1A with both D1 and D2 receptors facilitates dopamine transduction in vitro (Jiang et al. 2006; Kern et al. 2012). Furthermore, GHS-R1A is coexpressed with D2 receptors in hypothalamic neurons and with D1 receptors in the hippocampus and striatum (Jiang et al. 2006; Kern et al. 2012), which would support the notion that the effects of JMV 2959 on ARS and PPI could be mediated via modulatory effects on dopamine signaling. Similar to the effects of JMV 2959 to increase %PPI and decrease ASR, previous studies have shown that atypical antipsychotics that modulate dopamine receptor activity such as aripirazole and clozapine as well as the D2 receptor antagonists such as haloperidol dose dependently increase %PPI and decrease ASR in the acoustic startle and prepulse inhibition paradigm (Depoortere et al. 1997; Fejgin et al. 2007).

In our study, we were not able to find any effects of ghrelin treatment on %PPI and ASR, which might suggest that the GHS-R1A rather than ghrelin has an important role in regulating ASR and PPI. Supportively, the GHS-R1A has been shown to be constitutively active and the GHS-R1A/D2 heterodimer allosterically modify D2-mediated calcium mobilization in the absence of the endogenous ligand ghrelin; effects that were blocked by both D2 and GHS-R1A antagonism (Holst et al. 2003; Kern et al. 2012). Furthermore, previous findings on alcohol intake and alcohol-induced reward also suggest GHS-R1A-mediated rather than circulating ghrelin-mediated involvement in the regulation of dopamine transduction (Jerlhag et al. 2009, 2011a, b, 2014).

In the present study, we found that treatment with JMV 2959 completely blocked the effects of PCP on %PPI. Phencyclidine, a noncompetitive antagonist of the *N*-methyl-d-aspartate (NMDA) receptor, is known to induce a state that closely resemble schizophrenia in humans, including both positive and negative symptoms as well as cognitive dysfunctions (Yesavage and Freman 1978; Javitt and Zukin 1991), and has previously been used to investigate behaviors associated with schizophrenia in experimental subjects. In animals, PCP and other noncompetitive antagonists of the NMDA receptor such as MK-801 are widely used to model aspects of the human disease, including sensorimotor-gating deficits (Geyer et al. 2001). Recent findings have shown that ghrelin treatment can enhance NMDA receptor signaling through intracellular phosphorylation of the NR1 subunits of the NMDA receptor via the cAMP/PKA pathway indicating that ghrelin, possibly through the GHS-R1A, may interact with NMDA receptor signaling (Isokawa 2013a, b). However, we did not see any potentiating effects of ghrelin on sub-threshold PCP treatment in %PPI responses indicating that ghrelin is not involved in PCP-induced deficits of sensorimotor gating. Supportively, no associations between ghrelin levels and schizophrenia have been found in humans (Tsai et al. 2011). The interaction between ghrelin (putatively via GHS-R1A signaling) and the NMDA receptor may still, however, partially explain the beneficial effects of JMV 2959 on PCP-induced disruption of the PPI response. There is strong evidence for an interaction between dopamine and glutamate signaling in schizophrenia (Carlsson and Carlsson 1990; Bakshi et al. 1994; Bakshi and Geyer 1995; Fejgin et al. 2007). Thus, it has been put forth that a hyperdopaminergic condition could be a result of cortical NMDA receptor hypofunction with reduced inhibition of midbrain brain dopamine neuron firing as a consequence that may precipitate positive symptoms (Kegeles et al. 2000). The abolition of PCP-induced deficits in PPI by JMV 2959 could thus, in addition to direct modulations at the NMDA receptor, also be a result of a stabilizing effect of JMV 2959 on dopamine signaling. The present findings are based on systemic administration and further investigation of the neuroanatomical regulation of gating mechanisms by GHSR1A signaling using parenchymal brain injections of GHSR1A ligands is needed. A deeper understanding of how GHS-R1A antagonists, such as JMV 2959, alter dopamine transduction and GHS-R1A heterodimerazation with dopamine receptors will also give a better understanding of the effects of central GHS-R1A signaling in behaviors in general and in schizophrenia and schizophrenia-related behaviors specifically.
